# Supplementary material for: Bayesian refinement of protein structures and ensembles against SAXS data using molecular dynamics
Source: PLoS Comput Biol. 2017 Oct 18;13(10):e1005800. doi: 10.1371/journal.pcbi.1005800 (PMC5662244; doi:10.1371/journal.pcbi.1005800)
Supplement: S1 Archive — (BZ2) [file pcbi.1005800.s011.tar.bz2 › gromacs_4.62_bayesian_saxs_refi/share/html/online/g_velacc.html]

g\_velacc


| g\_velacc Main Table of Contents |

**VERSION 4.6  
Sat 19 Jan 2013**

---

### Description

g\_velacc computes the velocity autocorrelation function.
When the -m option is used, the momentum autocorrelation
function is calculated.

With option -mol the velocity autocorrelation function of
molecules is calculated. In this case the index group should consist
of molecule numbers instead of atom numbers.

Be sure that your trajectory contains frames with velocity information
(i.e. nstvout was set in your original .mdp file),
and that the time interval between data collection points is
much shorter than the time scale of the autocorrelation.

### Files

| option | filename | type | description |
| --- | --- | --- | --- |
| **-f** | traj.trr | Input | Full precision trajectory: trr trj cpt |
| **-s** | topol.tpr | Input, Opt. | Structure+mass(db): tpr tpb tpa gro g96 pdb |
| **-n** | index.ndx | Input, Opt. | Index file |
| **-o** | vac.xvg | Output | xvgr/xmgr file |
| **-os** | spectrum.xvg | Output, Opt. | xvgr/xmgr file |

### Other options

| option | type | default | description |
| --- | --- | --- | --- |
| **-[no]h** | bool | no | Print help info and quit || **-[no]version** | bool | no | Print version info and quit || **-nice** | int | 19 | Set the nicelevel || **-b** | time | 0 | First frame (ps) to read from trajectory || **-e** | time | 0 | Last frame (ps) to read from trajectory || **-dt** | time | 0 | Only use frame when t MOD dt = first time (ps) || **-[no]w** | bool | no | View output .xvg, .xpm, .eps and .pdb files || **-xvg** | enum | xmgrace | xvg plot formatting: xmgrace, xmgr or none || **-[no]m** | bool | no | Calculate the momentum autocorrelation function || **-[no]recip** | bool | yes | Use cm^-1 on X-axis instead of 1/ps for spectra. || **-[no]mol** | bool | no | Calculate the velocity acf of molecules || **-acflen** | int | -1 | Length of the ACF, default is half the number of frames || **-[no]normalize** | bool | yes | Normalize ACF || **-P** | enum | 0 | Order of Legendre polynomial for ACF (0 indicates none): 0, 1, 2 or 3 || **-fitfn** | enum | none | Fit function: none, exp, aexp, exp\_exp, vac, exp5, exp7, exp9 or erffit || **-ncskip** | int | 0 | Skip this many points in the output file of correlation functions || **-beginfit** | real | 0 | Time where to begin the exponential fit of the correlation function || **-endfit** | real | -1 | Time where to end the exponential fit of the correlation function, -1 is until the end |

---

http://www.gromacs.org  
gromacs@gromacs.org
